# Evaluation of GeneXpert MTB/RIF system performances in the diagnosis of extrapulmonary tuberculosis

**DOI:** 10.1186/s12879-019-4687-7

**Published:** 2019-12-19

**Authors:** Youness Mechal, Elmostafa Benaissa, Nadia El mrimar, Yassine Benlahlou, Fatna Bssaibis, Adil Zegmout, Mariama Chadli, Yashpal S. Malik, Nadia Touil, Ahmed Abid, Adil Maleb, Mostafa Elouennass

**Affiliations:** 1 Epidemiology and bacterial resistance research team/BIO-INOVA Centre, Faculty of Medicine and Pharmacy (University Mohammed V), Rabat, Morocco; 2 Department of Bacteriology, Mohammed V Military Teaching Hospital / Faculty of Medicine and Pharmacy (University Mohammed V), Rabat, Morocco; 3 Pneumology Department, Mohammed V University Hospital / Faculty of Medicine and Pharmacy (University Mohammed V), Rabat, Morocco; 4 Indian Veterinary Research Institute (IVRI), Bareilly, Uttar Pradesh India; 5 Research and Biosafety Laboratory, Mohammed V Military Teaching Hospital / Faculty of Medicine and Pharmacy (University Mohammed V), Rabat, Morocco; 6 Laboratory of Microbiology, Mohammed VI University Hospital / Faculty of Medicine and Pharmacy (University Mohammed the first), Oujda, Morocco

**Keywords:** Tuberculosis, Extra-pulmonary tuberculosis, Multidrug-resistance, Molecular diagnostic techniques, Morocco

## Abstract

**Background:**

Tuberculosis represents a serious public health problem and a significant diagnostic and therapeutic challenge worldwide. Molecular diagnostic techniques are crucial in the World Health Organization’s new tuberculosis control strategy.

This study aims to evaluate the performance of GeneXpert MTB/RIF (Cepheid Sunnyvale, CA, United States) in diagnosis of extra-pulmonary tuberculosis then compare it’s performance in detecting Rifampicin resistance to GenoType MTBDRplus (HAIN Life Sciences, Nehren, Germany).

**Methods:**

Samples from pulmonary and/or extra-pulmonary origins were analysed in a 21 months retrospective study. Samples were sent to the bacteriology laboratory for *Mycobacterium tuberculosis* detection using conventional bacteriological and molecular methods (GeneXpert MTB/RIF and MTBDRplus). Sensitivity and specificity were calculated for the stained smear and GeneXpert according to culture (Gold Standard) as well as for GeneXpert MTB/RIF in both negative and positive microscopy tuberculosis cases. Data’s statistical analysis was performed with SPSS13.0 software.

**Results:**

Seven hundred fourteen patients’ samples were analysed; the average age was 47.21 ± 19.98 years with a male predominance (66.4%). Out of 714 samples: 285 were from pulmonary and 429 were from extra-pulmonary origins. The positivity rates for microscopy, GeneXpert MTB/RIF and culture were 12.88, 20.59 and 15.82%, respectively. These rates were 18.9, 23.85 and 20.35% for pulmonary samples and 9.71, 18.41 and 12.82% for extra-pulmonary samples, respectively. The sensitivity and specificity of GeneXpert MTB/RIF were almost the same in both pulmonary and extra-pulmonary samples (78.2 and 90.4%) and (79,3 and 90.3%) respectively.

Rifampicin resistance rate found by GeneXpert MTB/RIF was 0.84%. Comparison of Rifampicin resistance obtained by GeneXpert MTB/RIF and Genotype MTBDRplus, showed 100% agreement between the two techniques for studied samples.

**Conclusions:**

This confirms GeneXpert MTB/RIF advantage for tuberculosis diagnosis, particularly extra-pulmonary tuberculosis with negatively stained smear. The performance of GeneXpert and Genotype MTBDRplus are similar in detection of Rifampicin resistance. However, variability of detection performance according to tuberculosis endemicity deserves more attention in the choice of screening techniques of Rifampicin resistance, hence the interest of conducting comparative studies of detection performance under low and medium endemicity on large samples of tuberculosis populations.

## Background

Tuberculosis is a global public health problem. The World Health Organization (WHO) reported 10.4 million new cases of tuberculosis and 1.7 million deaths (including 0.4 million co-infected with HIV) during 2016 [1]. Where 90% of the cases were from adults (65% male and 35% women), and 10% fromchildren. Notably, among the new cases recorded, 10% of the people are living with HIV, and 74% of whom are living in African countries. Further of the note, five countries, including India, Indonesia, China, Philippines and Pakistanshared 56% of all new cases recorded in 2016 [1]. The proportion of extra-pulmonary forms of tuberculosis reported by WHO in 2017 worldwide was estimated at 14% [2]. For anti-bacillary resistance, WHO estimates a Rifampicin resistance rate of 3.5% in new cases while 18% in treated cases. Moreover, the rates vary according to the region and tubercular endemicity [2].

In Morocco, 30,897 new cases of tuberculosis were recorded in 2017 (the case number was 30,636 in 2015) and rapid molecular biology technique “GeneXpert MTB/RIF” was employed only on 3% of the cases [3]. The incidence of tuberculosis in Morocco was 99/100,000 inhabitants during 2017, while the incidence of multidrug-resistant or Rifampicin-resistant tuberculosis was estimated at 1.5/100,000 inhabitants [3]. Extra-pulmonary form among the new cases (including relapses) in 2017 was 48%. Vis-a-vis tuberculosis resistance to anti-bacillary agents,the WHO reports a Rifampin resistance rate of 1% in new cases and 11% in previously treated cases in Morocco [3].

As of now, the diagnosis of extra-pulmonary tuberculosis remains challenging for both the clinicians and microbiologists globally. On the one hand, the extra-pulmonary tuberculosis is not always obvious to suspect during clinical examinations due to the variability of its clinical presentations [4]. On the other hand, the difficulty of access to specific sampling sites results in paucibacillary samples, which reduces the sensitivity of conventional diagnostic tests. The advent of molecular tests seems to bring a considerable gain in the diagnosis of extra-pulmonary tuberculosis, especially in the case of paucibacillary samples [4, 5].

Currently, the diagnosis of tuberculosis and anti-bacillary resistance relies on the phenotypic methods that evaluate the bacterial growth of different dilutions of mycobacteria in solid or liquid media in the presence of known concentrations of anti-bacillaries. However, these phenotypic methods have disadvantages such as operator dependency, the need for specialized laboratories and the long delay in reporting results (up to 10 days for solid medium techniques), and have a high cost of realization. Conversely, the adoption of molecular diagnostic tests for anti-bacillary resistance makes it possible to reduce the time-to-results and additively increasesthe diagnostic sensitivity by directly searching for mutations in the genes that determine resistance to different anti-bacillaries [6, 7].

The available molecular tests are GeneXpert MTB/RIF (Cepheid, Sunnyvale, CA, USA) and the Reverse Hybridization Test on strips GenoType MTBDRplus (HAIN Life Sciences, Nehren, Germany). GeneXpert MTB/RIF is currently the only rapid molecular test recommended by WHO for the rapid diagnosis of tuberculosis [2, 8]. GeneXpert MTB/RIF can detect both the presence of the *Mycobacterium tuberculosis* complex genome in patient specimens and the presence of genomic sequences of the main mutations responsible for Rifampicin resistance (rpoB gene mutation). The time limit for reporting the result is 2 h [9]. MTBDRplus is a multiplex DNA amplification test coupled with hybridization on strips for routine identification of mycobacteria and detection of genomic sequences of anti-tuberculosis drug resistance. The result can be reported within a few hours and allows the *Mycobacterium tuberculosis* complex and Rifampicin and Isoniazid resistance status to be detected in a single test [10].

The implementation of rapid molecular techniques for the diagnosis of tuberculosis is considered by WHO to be a significant asset in tuberculosis control strategies for the diagnosis and monitoring of tuberculosis disease [8, 11]. Scientific publications on the performance of GeneXpert MTB/RIF and Genotype MTBDRplus in detecting Rifampicin resistance report variable results depending on the regions in which these studies were conducted and the corresponding endemicity of tuberculosis [12, 13].

The objectives of this study were to evaluate the performance of GeneXpert in the diagnosis of extra-pulmonary tuberculosis in a tuberculosis-endemic country and then to compare the performance of GeneXpert MTB/RIF (Cepheid, Sunnyvale, CA, USA) and GenoType MTBDRplus in detecting Rifampicin resistance.

## Methods

In this retrospective study of 21 months sample data from pulmonary or extra-pulmonary origins from January 2017 to September 2018 was analysed. The samples were sent to the bacteriology laboratory for the detection of *Mycobacterium tuberculosis* by conventional bacteriological methods (microscopy and bacteriological culture) and by GeneXpert MTB/RIF. Additionally, patient data related to gender and age as well as tuberculosis diagnosis results by direct examination, culture and molecular biology techniques was collected.

The pulmonary samples (sputum, bronchial aspirations, bronchoalveolar lavage and protected distal sampling) and extra-pulmonary samples (cerebrospinal fluid, ganglion samples, tissue biopsies, osteoarticular sampling, pus, pleural punctures or biopsies, urinary samples and other localizations) were included in the study. The pathological samples were handled in a Biosafety level 2 laboratory (BSL-2).

### Sample processing & techniques of of *M. Tuberculosis* detection

#### Pulmonary samples (i.e. sputum, bronchial aspirations, bronchoalveolar lavage and protected distal sampling)

Samples were transferred to a 50 mark of falcon tube and concentrated by centrifugation at 3000 g for 15 min. Supernatants were discarded into a container with sodium hypochlorite containing 10% chlorine. Sediments were resuspended into 3–5 ml of sterile distilled water and were used for culture of *M. tuberculosis* as per the Modified Petroff’s method. In brief, the concentrate was digested and decontaminated using the sodium hydroxide (modified Petroff). The deposit (200 μl) was inoculated on two slopes of Lowenstein-Jensen (LJ) medium and incubated at 37 °C [14]. All slopes were observed for occurrence of growth daily for the first week and then twice weekly intervals for 8 weeks. Deficiency of growth at the end of the 8th week was regarded as a negative culture.

For Microscopy examination, concentrated smears after decontamination were examined according to the Ziehl-Neelsen and Fluorochrome (auramine O) staining techniques recommended by World Health Organization (1998) under a light or Fluorescence microscopes respectively. The entire length of the smear (2 cm) or 300 fields with light microscope using 1000×, were scanned. In case of an auramine slide with a fluorescent microscope, the entire length is also used while using 200x. A negative smear or examination is reported for those samples where no organisms observed.

#### Extra-pulmonary samples except CSF

Extrapulmonary specimens were divided into two main groups according to the extent of contamination. Aseptically collected tissues and contaminated specimens. Liquids were first concentrated for 15 min at 3000 g and sediments were resuspended in 2-5 ml of sterile distilled water. Biopsy (gonglionic specimen, tissue biopsies and other surgically resected tissue) were cut into small pieces with a sterile scalpel or scissors. They were homogenised in a sterile porcelain mortar and/or sonicated using 5 ml sterile saline buffer in the presence of 1-mm sterile glass beads.

For sterile samples, a portion (200 μl) without decontamination was inoculated into LJ medium, while the other portion was decontaminated by the N-acetyl-Lcysteine-sodium hydroxide (NALC-NaOH) method subjected to smear examination, LJ and a liquid medium using the BACTEC MGIT 960 system (BD Microbiology Systems) inoculation.

Contaminated specimens were preceded for culture of *M. tuberculosis* as per the Modified Petroff’s method and smear microscopic examination. Also, the method of NALC-NaOH decontamination for culture of *M. tuberculosis* in the MGIT liquid medium was used.

#### CSF

The collected CSF specimen was centrifuged and inoculated directly onto both liquid and solid media as described above. The smear examination is also performed.

### GenXpertTM or Xpert MTB/RIF assay

The Xpert MTB/RIF protocol for processed sample was tested as recommended by the manufacturer of GenXpertTMMTB/RIF, Cepheid (2009 April). Briefly, the proceeded samples were diluted with sample reagent (SR) at a ratio of 1:2. The sample/sample reagent mixture was shaken for at least 10s and incubated at room temperature for a total of 10 min. Recommended volume of the digested mixture was then transferred to the Xpert MTB/RIF cartridge and the automated steps of the procedure were started immeditaely after adding the sample to the cartridge.

### Genotype MTBDR*plu*s assay

Samples were screened using the genotype MTBDR*plus* assay according to manufacturer’s instructions [15]. Testing consisted of three steps: DNA extraction, multiplex PCR amplification using biotinylated primers and reverse hybridization. The three steps were carried out in three separated rooms. For DNA extraction, the Genolyse kit (Hain Lifescience, Nehren, Germany) and Nucelospin Genomic DNA from tissue kit (Macherey-Nagel) were used for pulmonary samples and extra -pulmonary samples respectively. Final DNA obtained was subjected to amplification in a classic thermocycler (MyCycler, BioRad). The run was considered valid if conjugate control and amplification control bands are present. A positive *Mycobacterium tuberculosis* control (TUB) band indicated the presence of members of the *M. tuberculosis* complex in the analysed sample.

### Staistical analyses

The data collection was done with Microsoft® Excel® 2007 spreadsheet, and statistical data analysis was performed with SPSS (IBM SPSS Statistics for Windows, Version 13.0) software. The study does not require a human research requiring Institutional Review Board approval.

Sensitivity, specificity and predictive values were calculated, by considering bacteriological culture as gold standard, using the 2 × 2 crosstab method on the SPSS software.

The positive and negative predictive values of GeneXpert MTB/RIF test were calculated only for the sample categories having a statistically significant size. Furthermore, comparison of the Rifampicin resistance detection performance between GeneXpert MTB/RIF and MTBDRplus was done.

## Results

In this study, a total of 714 patient samples were analysed. The mean age of patients was 47.21 ± 19.98 years with a male predominance accounted for 66.4%. The age distribution of patients was 3.9% for those under 15 years old, 74.9% for those between 15 andF 65 years old and 18% for those over 65 years old. The pulmonary samples were 285 out of the 714 samples (197 sputum, 73 bronchial aspirations, 08 bronchoalveolar lavage fluids and seven protected distal samples), and 429 samples were from extra-pulmonary origin (57 cerebrospinal fluids, 99 ganglion samples, 39 tissue biopsies, 27 osteoarticular samples, 112 pus, 65 pleural samples, nine urine samples, and 21 other miscellaneous samples).

**Fig. 1:**
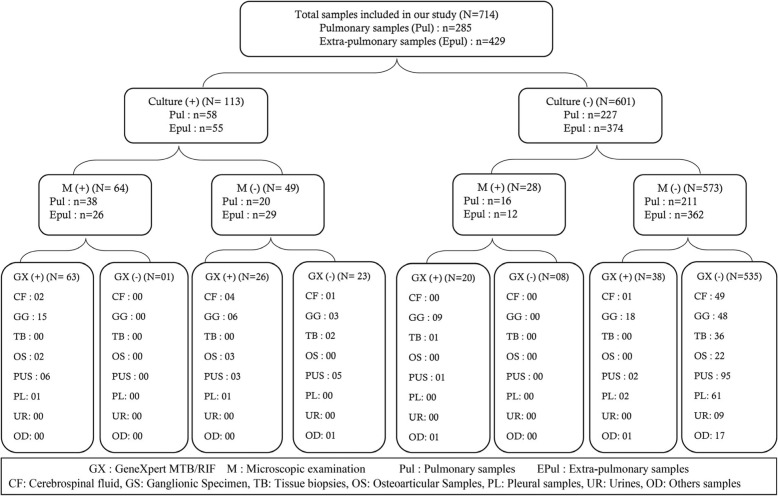
Distribution of samples included in our study by type and by sampling site

**Fig. 2:**
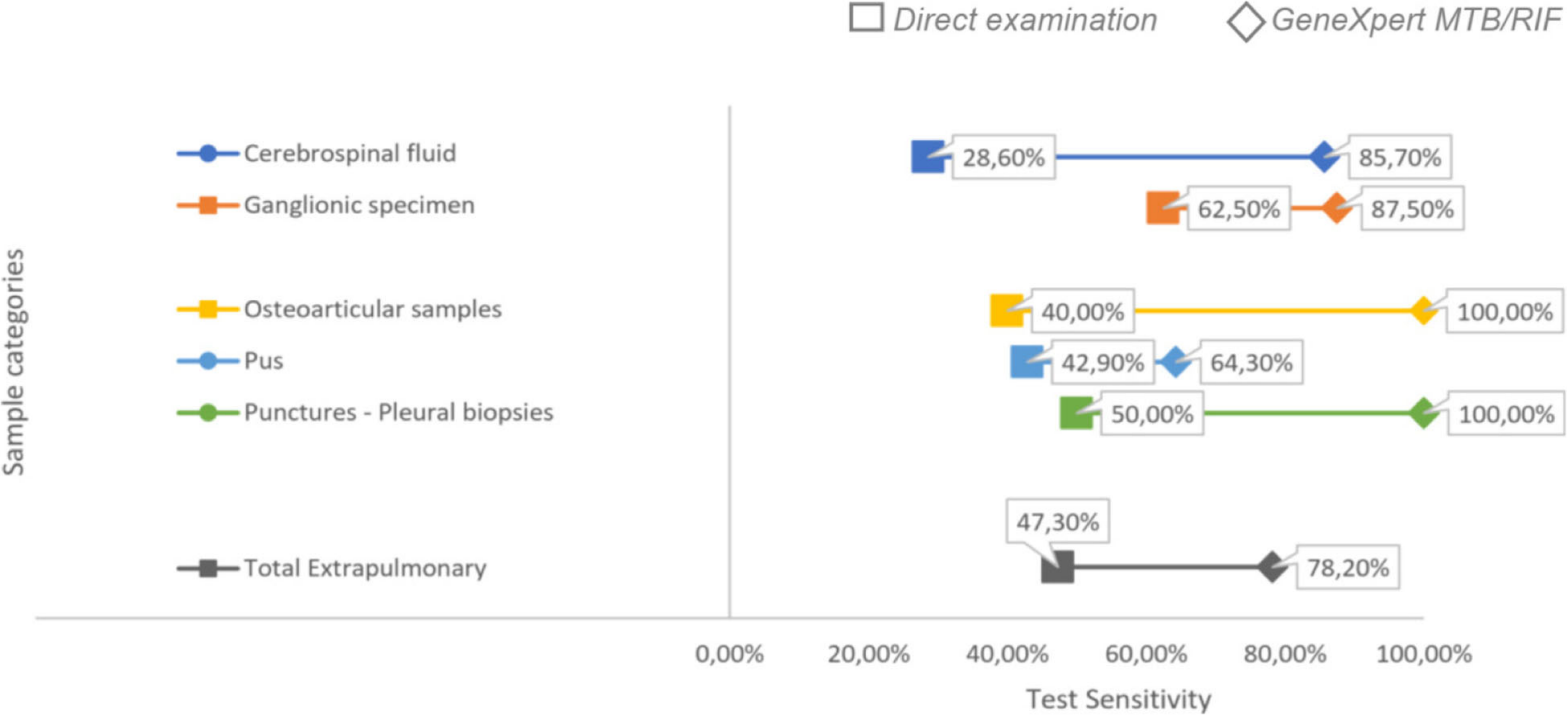
Sensitivities of direct examination and GeneXpert MTB/RIF in extra-pulmonary samples

Of the 714 samples tested by GeneXpert MTB/RIF, 147 samples (20.59%) were detected to be positive, and 567 (79.41%) were found to be negative. The results of microscopy, GeneXpert MTB/RIF and culture for all samples are summarized in Fig. 1. The positivity rates for microscopy, GeneXpert MTB/RIF and culture were 12.88, 20.59 and 15.82%, respectively. These rates were 18.9, 23.85 and 20.35% for pulmonary samples and 9.71, 18.41 and 12.82% for extra-pulmonary samples, respectively. Among the negative culture results, 58 samples (9.65%) had a positive result for GenXpert MTB/RIF where lymph node sampling represented 46.55% of cases (27 samples). In negative cultures, with a positive result for GenXpert MTB/RIF: 20 samples (34.48%) also had a positive microscopy result of which 9 (45%) were lymph node samples. The results of microscopy, GeneXpert MTB/RIF and culture for all samples are summarized in Fig. 1. The sensitivity and specificity profiles of microscopy and GeneXpert MTB/RIF in comparison to culture (reference technique), with an estimation of positive and negative predictive values, for each of the sample categories, are summarised in Table 1 and Fig. 2.

**Table 1:**
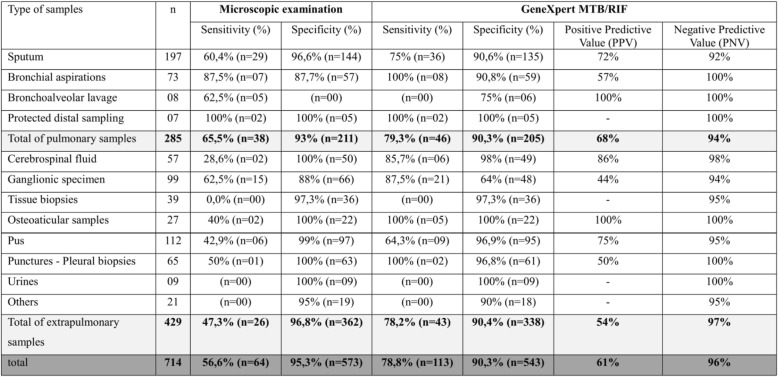
Diagnostic Performance parameters of the direct examination and GeneXpert by sample type

Diagnostic results for Rifampicin resistance by GeneXpert MTB/RIF on the 714 samples analysed showed a Rifampicin resistance rate of 0.84% (*n* = 6). The comparison of Rifampicin resistance results obtained by GeneXpert MTB/RIF and Genotype MTBDRplus, for a total of 43 samples showed a 100% agreement between the two techniques for six samples.

## Discussion

The main finding of this study is that extra pulmonary samples reveal the high sensitivity and the specificity of GeneXpert MTB/RIF for the diagnosis of extrapulmonary tuberculosis as compared to those found for pulmonary specimes. However, these rates vary according to the sampling site. Thus, the sensitivity of the GeneXpert MTB/RIF vary in our series between 64.3% in pus samples and 100% in osteoarticular samples. The specificity was 64% in ganglion samples and 100% in osteoarticular samples. This low specificity rate in ganglion samples can be explained by the number of tuberculosis cases with negative cultures. Indeed, among the 27 negative cultures of ganglion specimens, with GeneXpert MTB/RIF positive, nine samples were found to be positive for microscopic examination.

Taking into account the result of the microscopy, the specificity of GeneXpert MTB/RIF in ganglion specimens increased up to 72.7% while the sensitivity was 90.9%. The negativity of the culture, for this sampling category, could be related to the quality of the ganglion samples (dilution, transport and preparation of lymph node tissues) and the taking of antibiotics such as fluoroquinolones or anti-bacillaries before sampling. The high sensitivity and specificity values for osteoarticular specimens can be explicated by the fact that for this category the diagnosis of tuberculosis is most often considered as a last resort after antibiotic therapy-based treatment failure and therefore these are patients with a very high suspicion of osteoarticular tuberculosis.

Another interesting finding is that GeneXpert MTB/RIF performances, based on the total (pulomonary and extrapulmonary) results of the microscopic examination has 20.6% more sensitivity than microscopic examination. This increase was highlighted for extrpulmonary samples (30%).

This performance of GeneXpert MTB/RIF evaluated on samples from extrapulmonary origins is considerable for the clinicians who could manage the patient more quickly and reduce the epidemiological risks. The clinicians could rapidly implement the anti-tuberculosis drug without having to wait for the results of the culture in case of a negative microscopic examination. Shiying Li et al. (2017) found in the meta-analysis that he conducted on studies from different regions corresponding to different degrees of tubercular endemicity, a sensitivity ranging from 97 to 99% in positive microscopy samples versus 68 to 73% in negative microscopy samples. Opota et al. (2019) found a sensitivity of 100% for positive microscopy samples versus 66,67% for negative microscopy samples [16, 17].

Our results are in line with those obtained in countries with similar tuberculosis endemicity [18–20]. Indeed, the performance obtained through similar studies varies according to the tubercular endemicity and the nature of the site sampled. In countries with low endemicity rates, the sensitivity and the specificity of GeneXpert MTB/RIF varies between 82,98 and 95% for sensitivity, and between 96 and 99% for specificity. However, in countries with high endemicity, these rates vary between 80 and 88% for sensitivity, and between 95 and 98% for specificity [16, 17, 21–24].

Among negative culture samples, 10.61% were found to be positive for GeneXpert MTB/RIF. Similar findings were observed by Pandey and Iram with 11 and 15%, respectively [10.25]. This could be due to the nature and quality of the samples received in terms of their richness in tubercle bacilli, the sampling methods used, and the antibiotic or anti-bacillary treatments taken preceding the sampling.

The negative predictive value obtained for pulmonary and extra-pulmonary samples (94 and 97% respectively) indicates the interest of GeneXpert MTB/RIF in eliminating the tuberculosis. This is in line with WHO data on GeneXpert MTB/RIF for Positive predictive value (PPV) and Negative predictive value (NPV). According to WHO, the NPV of GeneXpert MTB/RIF exceeds 99% regardless of the tuberculosis prevalence rate makingit possible to exclude with assurance the diagnosis of tuberculosis. The PPV rate is negatively influenced by the decrease in tuberculosis prevalence from 94% (for a tuberculosis prevalence of 15%) to 65% (for a tuberculosis prevalence of 2%) [15].

All the results of current study are in line with the WHO recommendations on tuberculosis diagnosis, which highlights the importance of molecular research for the entire population of tuberculosis suspects, especially for the high risk groups such as suspected multidrug-resistant tuberculosis and suspected HIV-related tuberculosis. The current findings confirm the relevance of WHO’s recommendations to make the molecular diagnosis by GeneXpert MTB/RIF as a main diagnostic approach [15].

The overall resistance rate to Rifampin in our series was 0.84%, which is close to the WHO estimate for Morocco for primary resistance of 1% (established on a 4% reporting basis). The Isoniazid (INH) resistance rate in our study is 10.34% in samples tested by MTBDRplus Genotype and is in accordance with regional study data where El Baghdadi et al. found an isoniazid resistance rate of 11.4% in the region of Casablanca. Karimi et al. found a resistance rate of 17.1% in the Tangier region [25, 26]. The comparison of the rifampicin resistance performance results in current study by MTBDRplus Genotype with those obtained by GeneXpert MTB/RIF shows a 100% agreement for the 43 samples analysed. These results are consistent with those obtained by Rahman et al. [12] where 92.4% agreement between the two molecular tests was found.

The concordance rate decreases sharply in studies from countries with high tuberculosis endemicity like 61.4% in India as reported by Rufai et al. The latter study showed that the results obtained by GeneXpert MTB/RIF were associated with false positives and false negatives of 5.1 and 33.8%, respectively [13]. False positive Rifampicinresistance by GeneXpert MTB/RIF is often linked to a low detection level (paucibacterial sampling) as well as to certain mutations in the rpoB gene that is the target of GeneXpert MTB/RIF detection probes [27]. The recognition of the non-functional rpoB F514F gene as a rifampin resistance gene has also been described [28].

The studies conducted by Blakemore et al. and Zetola et al. associates the false negative results of GeneXpert MTB/RIF for Rifampin resistance detection with the presence of a mix of sensitive and resistant bacilli in the same sample. This situation is frequently encountered in highly endemic countries [29, 30]. Poor detection performance of GeneXpert MTB/RIF is also described in a study by Rufai et al. [13]. Notably, 5% of Rifampin resistance is due to mutations outside the rpoB gene and is consequently not detected by the GeneXpert MTB/RIF [29].

This performance determination of GeneXpert MTB/RIF seems to be related to the nature of the probes used for the detection of mutations responsible for Rifampin resistance, especially the E probe which has a hybridization rate of only 52% in a very highly endemic country like India [13]. The adjustment of detection probes according to the predominant mutations in each region seems essential for the reliability of GeneXpert MTB/RIF for the detection of rifampicin resistance.

This limit of GeneXpert MTB/RIF requires, in regions of high tubercular endemicity, to confirm resistance results obtained by a confirmation test such as Genotype MTBDRplus or MGIT960 DST which have high detection performance (using LJ-DST as the gold standard) [13]. This confirmation requirement has been recommended by the Centers for Disease Control and Prevention (CDC) [28].

Worldwide, tuberculosis resistance to anti-bacillary treatments was estimated by WHO in 2017 at 18% in treated cases and 3.5% in new cases. For the African continent, this ratewas 14% in the treated cases and 2.7% in the new cases [2]. In Morocco, the WHO estimates the resistance rate at 1% in new cases and 8.7% in treated cases. However, this estimation is based only on the reporting of rifampin resistance tests for 4% of tuberculosis cases. Whereas the global reporting rate was 24% [2, 3, 31].

Rapid molecular diagnosis (GeneXpert MTB/RIF), allowing both the diagnosis of tuberculosis and its resistance to anti-bacillary agents, have been used in only 3% of reported tuberculosis cases (530 cases) [3].

The WHO recommended GeneXpert MTB/RIF in 2010 for the diagnosis of pulmonary tuberculosis and subsequently in 2013 for the diagnosis of extra-pulmonary tuberculosis [2]. WHO recommendations for the integration of GeneXpert MTB/RIF in the tuberculosis diagnosis process are linked to its short time to results and demonstrated performance (sensitivity and specificity) for both pulmonary and extra-pulmonary tuberculosis diagnosis [32].

The WHO has inititated a tuberculosis control strategy to end the tuberculosis epidemics by 2035 [33].

## Conclusions

The findings of this study confirm GeneXpert MTB/RIF as a test of choice for the diagnosis of extra-pulmonary tuberculosis due to its high sensitivity and specificity performances. Its interest is also highlighted in cases of tuberculosis with negative microscopic examination where it considerably increases the sensitivity of the diagnosis and the early medical management of patients in line with WHO recommendations. The results encourage the integration of GeneXpert MTB/RIF into tuberculosis control programs. The interest of GeneXpert MTB/RIF in assessing anti-bacillary susceptibility depends on the tuberculosis incidence rate. This interest is evident in countries with low tuberculosis incidence with high sensitivity (> 90%), and decreases in regions with very high endemicity where the sensitivity decreases up to 50%. Furthermore, in all cases, the sensitivity results obtained by GeneXpert MTB/RIF may be checked by another molecular diagnostic test as recommended by Centers for Disease Control and Prevention.

## Data Availability

The datasets used and analysed during the current study are available from the corresponding author on reasonable request.

## Abbreviations

CDC: Centers for Disease Control and Prevention
HIV: Human immunodeficiency virus
INH: Isoniazid
LJ: Lowenstein-Jensen
NPV: Negative predictive value
PPV: Positive predictive value
WHO: World Health Organization
